# A Bivalent mRNA Vaccine Efficiently Prevents Gammaherpesvirus Latent Infection

**DOI:** 10.3390/vaccines13080830

**Published:** 2025-08-04

**Authors:** Yannan Yin, Jinkai Zang, Huichun Shi, Zhuang Wang, Linlin Kuang, Shuxia Wang, Haikun Wang, Ning Li, Xiaozhen Liang, Zhong Huang

**Affiliations:** 1 Shanghai Institute of Immunity and Infection, Chinese Academy of Sciences, University of Chinese Academy of Sciences, Shanghai 200031, China; ynyin@siii.cas.cn (Y.Y.); llkuang@siii.cas.cn (L.K.); 2 Shanghai Institute of Infectious Disease and Biosecurity, Fudan University, Shanghai 200032, China; jkzang0708@gmail.com (J.Z.); hcshi19@fudan.edu.cn (H.S.); 23111020018@m.fudan.edu.cn (Z.W.); shuxia_wang@fudan.edu.cn (S.W.); 3 State Key Laboratory of Cardiovascular Diseases, Shanghai East Hospital, School of Medicine, Tongji University, Shanghai 200092, China; hkwang@tongji.edu.cn; 4 Shanghai Key Laboratory of Infectious Diseases and Biosafety Emergency Response, Department of Infectious Diseases, National Medical Center for Infectious Diseases, Huashan Hospital, Shanghai Medical College, Fudan University, Shanghai 200040, China

**Keywords:** murine gammaherpesvirus 68 (MHV68), mRNA vaccine, neutralizing antibody, T-cell response, latent infection

## Abstract

**Background**: It is still challenging to develop effective vaccines against tumorigenic human gammaherpesviruses such as Epstein–Barr virus (EBV). A major obstacle is the lack of a small animal model that reproduces the natural infection course of human gammaherpesviruses to allow for proper assessment of vaccine efficacy. Murine gammaherpesvirus 68 (MHV68) is a natural pathogen of wild rodents and laboratory mice and therefore can be used as a surrogate for human gammaherpesviruses to evaluate vaccination strategies. **Methods**: In this study, two mRNA vaccine candidates were generated, one encoding a fusion protein of the MHV68 gH with the gL (gHgL-mRNA) and the other expressing the MHV68 gB protein (gB-mRNA). The immunogenicity and protective efficacy of the mRNA vaccine candidates were evaluated in a mouse model of MHV68 infection. **Results**: The gHgL-mRNA but not the gB-mRNA candidate vaccine was able to induce neutralizing antibodies in mice, whereas both vaccines could elicit antigen-specific T-cell responses. Following MHV68 intranasal inoculation, complete blocking of the establishment of viral latency was observed in some mice immunized with individual gHgL-mRNA or gB-mRNA vaccines. Notably, co-immunization with the two mRNA vaccines appeared to be more effective than individual vaccines, achieving sterile immunity in 50% of the vaccinated mice. **Conclusions**: This study demonstrates that immunization with mRNA platform-based subunit vaccines is indeed capable of preventing MHV68 latent infection, thus validating a safe and efficacious vaccination strategy that may be applicable to human gammaherpesviruses.

## 1. Introduction

Human gammaherpesviruses, exemplified by Epstein–Barr virus (EBV), are a group of pathogens that may cause severe clinical consequences. In particular, EBV infection could lead to infectious mononucleosis (IM) and multiple lymphoid and epithelial cancers, such as Burkitt and Hodgkin lymphomas, gastric carcinoma, and nasopharyngeal carcinoma [1,2,3,4]. Furthermore, EBV infection is associated with multiple sclerosis (MS), a potentially disabling chronic disease that affects over 900,000 Americans and 2.8 million people worldwide [5,6]. However, there is no vaccine available for preventing EBV infection. EBV is usually transmitted by saliva and contracted through kissing, coughing, or sharing food and eating utensils [7,8,9]. Following transmission, EBV establishes primary infection in oral epithelial cells, wherein productive virus replication takes place. Another cell type targeted by EBV is B lymphocyte, which is where EBV infection progresses into the latency phase [10,11]. EBV maintains a latent state primarily in memory B cells, enabling long-term persistence and potential reactivation. Humans are the only natural host for EBV. Transfer of human hematopoietic stem cells into NOG mice can yield humanized mice that permit EBV infection of B cells via intravenous injection, but such a model does not reproduce the natural infection course, especially the first stage-infection of epithelial cells [12,13]. Thus, the absence of a small animal model that can mimic the natural EBV infection has been one of the major impediments to the development and evaluation of EBV vaccines.

*Murine gammaherpesvirus 68* (MHV68) is a close relative to EBV. Both viruses display remarkable genetic similarities in their genomic architecture and functional elements, with 80% of their genes being homologous [14,15,16]. The essential genes for viral entry, such as glycoprotein B (gB) and gH/gL, exhibit functional and sequence conservation between MHV68 and EBV, indicating their shared strategies for invading host cells [17,18]. MHV68 is able to infect a variety of small rodents, including laboratory mice [19,20,21]. After mice were intranasally inoculated with MHV68, an acute infection was established in lung and nasal epithelial cells, followed by a transition into latency within B cells, particularly in the spleen and other lymphoid tissues of infected mice. During latency, the viral genome persists in the host cell without active replication [22,23,24,25]. MHV68 infection may lead to lymphoproliferative diseases, characterized by increased proliferation and inflammatory responses in lymphoid tissues [26,27]. Clearly, MHV68 intranasal inoculation not only accurately replicates the mucosal infection that is characteristic of natural transmission of EBV, but also replicates a latency state established primarily in B cells and diseases akin to those seen with EBV infection in humans. As a result, MHV68 can be utilized as a substitute model to investigate the mechanisms behind the pathogenesis and immune control of EBV, and to assess the effectiveness of vaccination strategies [28,29].

Several types of MHV68 vaccine have been developed and evaluated [30], such as a live attenuated virus vaccine [31,32,33,34,35,36,37], an inactivated whole-virus vaccine [38], and a heterologous viral vector-based vaccine [39,40,41]. Only the replication-competent MHV68 virus mutants, in which either latency or immune evasion genes were deleted, were able to prevent viral latency [32,33,34,35,36,42]; however, such a vaccine approach is unsuitable for human gammaherpesviruses like EBV due to its oncogenic nature. Previous work has shown that neutralizing antibodies against MHV68 mainly target the gH, gL, and gB proteins [43,44], suggesting that these proteins, as vaccine antigens, may have the potential to provide protection in vivo. In the present study, we generated mRNA vaccine candidates expressing a fusion protein of the gH and gL (hereinafter referred to as gHgL) or the gB protein of MHV68, and subsequently assessed their immunogenicity and protective efficacy. Both the gHgL- and the gB-expressing mRNA vaccines were found to provide protection against MHV68 latent infection in varying degrees in mice. Notably, the combination of the two mRNA vaccines was more effective than the individual vaccines in eliciting sterile immunity. These results demonstrate for the first time that subunit vaccines based on the mRNA platform are capable of preventing MHV68 latent infection, thus validating a new strategy to develop safe and effective vaccines against human gammaherpesviruses.

## 2. Materials and Methods

### 2.1. Viruses and Cells

All cell lines were maintained at 37 °C in a humidified air comprising 5% CO_2_. HEK 293T cells were cultured in DMEM (Gibco, Grand Island, NY, USA) supplemented with 10% fetal bovine serum (FBS; Gibco) and 1% penicillin–streptomycin. HEK 293F cells were cultured in FreeStyle 293 expression medium (Gibco). The NIH3T12 cell line was cultured in DMEM containing 10% FBS and 1% penicillin–streptomycin. MHV68-H2bYFP virus [45] was propagated in NIH3T12 cells as described previously [46]. Virus titers were determined by 50% tissue-culture infectious dose (TCID_50_) using NIH3T12.

### 2.2. Construction of mRNA Vaccine Plasmids

**Figure 1:**
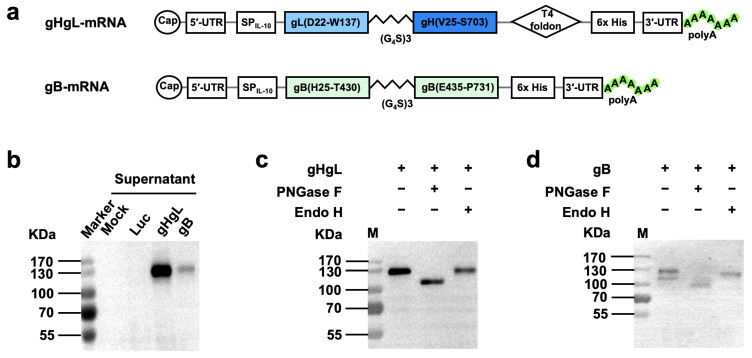
Construction and characterization of mRNA vaccine vectors. (**a**) Schematic diagram of gHgL (top panel) and gB (bottom panel) mRNA framework. MHV68 gL is fused to the N terminus of gH through a flexible amino acid linker, and a C-terminal T4 foldon is introduced. gB furin cleavage site is replaced by a flexible amino acid linker. UTR, untranslated region; SP_IL-10_, human interleukin-10 signal peptide; T4 foldon, T4 fibritin trimerization motif. (**b**) The culture supernatants of in vitro transcribed mRNA-transfected HEK293T cells were analyzed for gHgL and gB expression by Western blotting with HRP-conjugated anti-His-tag antibody. Luc, mRNA encoding luciferase. (**c**,**d**) Supernatants of gHgL mRNA-transfected cultures (**c**) and gB mRNA-transfected cultures (**d**) were digested with either endo H or PNGase F, and then subjected to Western blotting analysis with an HRP-conjugated anti-His tag antibody. Symbol (+) indicates presence; (−) indicates absence. M, protein marker.

The construction of mRNA vaccine plasmids was performed as previously described [47]. Briefly, cDNA encoding gL (residues D22 to W137), gH (residues V25 to S703), or gB (residues H25 to T430, E435-P731) was codon-optimized and -synthesized by Genscript (Shanghai, China). These fragments were fused with T7 promotor, 5′-untranslated region (5′-UTR:5′-AAATAAGAGAGAAAAGAAGAGTAAGAAGAAATATAAGAGCCACC-3′), interleukin-10 (IL-10) signal sequence, 3′-UTR (5′-TGATAATAGGCTGGAGCCTCGGTGGCCATGCTTCTTGCCCCTTGGGCCTCCCCCCAGCCCCTCCTCCCCTTCCTGCACCCGTACCCCCGTGGTCTTTGAATAAAGTCTGA-3′), and a poly-A tail of about 101 base pairs (bp) (Figure 1a). The 3′ end of the gH sequence was also linked with the folded trimerization domain sequence from T4 bacteriophage fibrin [48]. To facilitate verification of expression of the target proteins, a 6x His-tag coding sequence was appended in-frame to the 3′-end of the gH or gB sequences. Then, the assembled gHgL and gB expression cassettes were individually cloned into pUC57 vector, yielding the two final mRNA vaccine plasmids termed gHgL-mRNA and gB-mRNA, respectively (Figure 1a).

### 2.3. Recombinant Proteins and Antibodies

To express and purify recombinant gHgL protein in mammalian cell culture, the coding sequence for gHgL fused with a 6x His-tag was cloned into pcDNA3.4 backbone vector, yielding plasmid pcDNA3.4-gHgL. Then, HEK 293F cells were transfected with the plasmid pcDNA3.4-gHgL. Proteins were purified from the supernatant of transfected HEK 293F cell culture using Ni-NTA resin (Millipore). The resultant recombinant gHgL fusion protein was used as ELISA coating antigen in subsequent serum antibody measurement experiments.

To obtain recombinant gB protein, the coding sequence for gB-N (residues H25 to T430) fused with a 6x His-tag was cloned into pET-28b backbone vector, yielding pET-28b-gB-N. Then, the plasmid pET-28b-gB-N was transformed into *Escherichia coli* BL21 cells for prokaryotic expression. Proteins were purified from the lysate of the pET-28b-gB-N-transformed *E. coli* using Ni-NTA resin. The resultant recombinant gB-N protein was used as an ELISA coating antigen in subsequent serum antibody measurement experiments.

Anti-gB polyclonal antibody was generated from BALB/c mice immunized with *E. coli*-produced recombinant gB protein formulated with Freund’s adjuvant (Sigma-Aldrich, St. Louis, MO, USA). This polyclonal antibody serves as a positive control in ELISA assays.

### 2.4. mRNA and LNP Production

The mRNA vaccine plasmids were linearized by Not I digestion and used for in vitro mRNA generation, using the T7 RNA Transcription Enzyme Kit (Novoprotein, Suzhou, China; Catalog No. E131-01A). After purification, the mRNAs were capped, utilizing vaccinia capping system (New England BioLabs, Ipswich, MA, USA, M2080S) and mRNA Cap 2′ -OMethyltransferase (New England BioLabs, M0366S) to produce the Cap I structure. The capped mRNAs were purified and stored at −80 °C until use.

LNP was prepared as previously described [47], using four kinds of lipids, namely D-Lin-MC3-DMA (MedChemExpress Monmouth Junction, NJ, USA), DSPC (Avanti Polar Lipids, Alabaster, AL, USA), cholesterol (Sigma-Aldrich) and PEG-lipids (Avanti Polar Lipids). Then, the properly diluted capped mRNAs were mixed with LNP to achieve a final mRNA/lipid ratio of 0.056 mg/mmol. Subsequently, the LNP-mRNA mixtures were incubated for 30 min at 42 °C, dialyzed against PBS (pH 7.4) for more than 18 h, concentrated to a certain volume with a 3 KD ultrafiltration tube, filtered through a 0.45 filter membrane, and stored at 4 °C until use.

### 2.5. Expression of the mRNA in HEK293T Cells

When the HEK 293T cell density in the 12-well plate grew to 80–90%, the Lipofectamine 2000 Transfection Reagent (Invitrogen, Thermo Fisher Scientific, Waltham, MA, USA) was used to transfect in vitro capped mRNAs into cells, according to the manufacturer’s instructions. After 48 h, the transfected cells and culture supernatant were collected and analyzed by Western blot with HRP-conjugated anti-His tag antibody (Rosemont, IL, USA).

### 2.6. Mouse Immunization and Virus Challenge

All the animal experiments in this study were approved by the Institutional Animal Care and Use Committee at the Shanghai Institute of Immunity and Infection (Protocol Number: A2023009).

In the first immunization experiment, three groups of six female C57BL/6J mice (6–8 weeks old) were immunized intramuscularly (i.m.) with LNP-encapsulated gHgL or gB mRNA vaccines (10 μg of mRNA per dose), respectively, at week 0, and boosted with the same dose at weeks 3, 6, and 16. Another group of mice were injected with the Luc-mRNA-LNP formulation (10 μg of mRNA per dose), used as a control. Blood samples were collected for analysis of binding antibody and neutralizing antibody titers from individual mice at weeks 8 and 18. Three weeks after the fourth injection, mice were anesthetized with isoflurane and intranasally challenged with 10^5^ PFU of MHV68 viruses that were diluted in 20 μL of PBS. On day 16 post-challenge, the mice were euthanized, and spleens were immediately harvested from the euthanized animals. Individual spleens were ground into a single-cell suspension, and then the YFP-positive cells were measured by flow cytometry. For viral load measurement, DNA was extracted from 5 million splenocytes using the genomic DNA Kit (TIANGEN Biotech, Beijing, China).

In the second immunization experiment, three groups of four female C57BL/6J mice were immunized intramuscularly with LNP-encapsulated gHgL, gB, or Luc-mRNA vaccines (10 μg of mRNA per dose) at week 0, followed by a booster dose of the same formulation at week 2. One week after the booster immunization, spleens were harvested for ELISpot and flow cytometric analysis.

In the third immunization experiment, four groups of twelve female C57BL/6J mice were immunized intramuscularly with LNP-encapsulated gHgL, gB, gHgL/gB (containing 10 μg gHgL-mRNA and 10 μg gB-mRNA), or Luc-mRNA vaccines (10 μg of mRNA per dose) at week 0, followed by a booster dose of the same formulation at weeks 2 and 4. Blood samples were collected week 6. Three weeks after the third injection, eight mice were randomly selected from each group for the viral challenge. The spleens were harvested for analysis 16 days after the challenge for flow cytometry, qPCR, and Infectious center assay.

### 2.7. Quantitative PCR (qPCR)

The extracted spleen DNA was used for quantitative PCR. qPCR was performed with a SYBR^®^ Premix ExTaqTM kit (Takara Bio, Shiga, Japan), using primers located within the ORF59 coding sequence (ORF59: 5′-GGAGTGAAATATGTGGAGGCACAATTTGTGCA-3′ and 5′-TTATGGGAAGTGGTTAAAATGTAGC-3′). An MHV68 ORF59 DNA fragment was used as the reference standard of viral genome copy numbers in qPCR assays, and defined the limit of detection being 100 viral genome copies, as measurements below this threshold were found to be unreliable based on the reference standard. For statistical analysis, samples with no amplification or those yielding readings below the limit of detection were assigned a value of 100 viral genome copies.

### 2.8. Serum Antibody Measurement

For antibody titration, the 96-well microplates were coated with 100 ng/well of gHgL or gB proteins at 4 °C overnight, followed by blocking with 5% milk diluted in PBST for 1 h at 37 °C. Subsequently, the plates were incubated with serially diluted mouse antisera (50 μL/well) for 1 h at 37 °C and then incubated with properly diluted HRP-conjugated anti-mouse IgG (Sigma) for 1 h at 37 °C. After color development with HRP substrates, absorbance at 450 nm was measured. The binding IgG endpoint titer was defined as the highest serum dilution that had an absorbance over 0.1 optical density units above that of the Luc-group serum samples.

### 2.9. Neutralization Assay

Sera were heat-treated at 56 °C for 30 min prior to neutralization assays to inactivate the complement. 3T12 cells were seeded into 96-well microplates and incubated at 37 °C until confluent cell monolayers formed. Serially diluted serum in FBS-free DMEM was incubated with MHV68 at 37 °C for 1 h. The serum–virus mixtures were then added to 3T12 cells for a 1 h adsorption at 37 °C. Cells were supplemented with the same volume of 10% FBS-DMEM and then incubated for 48 h at 37 °C. After 48 h, the cell supernatants were discarded, and then the plates were scanned by Immunospot^®^ S6 Universal M2 Analyzer, and YFP-expressing cells were counted and analyzed.

### 2.10. Infectious Center Assay

Splenocytes were serially diluted, layered onto a Vero cell monolayer, and then incubated overnight at 37 °C. The splenocytes were discarded, and then the plates were washed. The Vero cells were overlaid with 0.8% (*w*/*v*) methylcellulose in DMEM containing 2% (*w*/*v*) FBS. Five days later, the plates were soaked in the 4% formaldehyde to fix cells, followed by staining the plate with 1% crystal violet. Plaques were counted to indicate infection centers (ICs). For graphical purposes, samples with no detectable infectious centers were assigned a default value of 1IC per 1 × 10^7^ cells to facilitate graphical representation.

### 2.11. Enzyme Linked Immunospot^®^ (ELISpot) Assay

The plates were precoated with Mouse IFN-γ ELISpot capture antibody (BD) overnight at 4 °C. The plates were blocked using RPMI 1640 containing 10% FBS at 37 °C for at least 3 h. Immunized mouse splenocytes were collected and then plated at 1 × 10^6^ cells/well, and they were stimulated with the gHgL-spytag protein or gB peptide (KNYIFEEKL; synthesized by GL Biochem, Shanghai, China)), Concanavalin A (ConA, Sigma-Aldrich) as a positive control, or DMSO as a negative control. After incubation at 37 °C and in 5% CO_2_ for 48 h, the cells and medium were decanted, and then the plates were washed with wash buffer, and biotin-conjugated mouse IFN-γ ELISpot detection antibodies were added to the plate, followed by incubation for 3 h at room temperature. AP-conjugated Streptavidin was added to each well, and plates were incubated for 45 min at room temperature, followed by the addition of BCIP/NBT color development substrate for 30 min. Finally, development was stopped by washing wells 3 times with ddH_2_O and air-drying in darkness overnight. The spots were read using Immunospot^®^ S6 Universal M2 Analyzer (CTL, Cleveland, OH, USA).

### 2.12. Intracellular Cytokine Staining (ICS) and Flow Cytometry

ICS assays were carried out as previously described [49], with minor modifications. Briefly, spleens were harvested from immunized mice to prepare splenocytes. About 2 × 10^6^ splenocytes were added to each well of 96-well U-plates. The gHgL-spytag proteins or gB peptide were added to splenocytes for stimulation overnight at 37 °C. The cells with or without addition of ionomycin and PMA (Phorbol 12-myristate 13-acetate) were set up as positive and negative controls, respectively. Three hours before cell collection, Brefeldin A was added to cells in order to inhibit cytokine secretion. Prior to staining, the splenocytes were incubated with Fc blocker. Then, the cells were stained with antibodies specific for mouse cell surface markers (including anti-CD4 labeled with FITC, anti-CD8 labeled with Pacific Blue, and anti-CD44 labeled with Brilliant Violet 605) and then the Live/Dead^®^ Fixable Dead Cell Stain (Invitrogen, Thermo Fisher Scientific). Subsequently, the cells were subjected to fixation and permeabilization, followed by staining with anti-IFN-γ labeled with PerCP-Cy5.5, anti-IL-2 labeled with PE, and anti-TNF-α labeled with PE-Cy7 antibodies. After staining, the samples were analyzed with a flow cytometer (BD LSRFortessa™, BD Biosciences, San Jose, CA, USA).

## 3. Results

### 3.1. Construction and Characterization of the MHV68 mRNA Vaccine Vectors

We constructed two MHV68 mRNA vaccine vectors, designated gHgL-mRNA and gB-mRNA (Figure 1a). The gHgL-mRNA vector was designed to express a fusion protein consisting of the IL-10 signal peptide, the MHV68 gL protein, a flexible (Gly_4_Ser_1_)_3_ linker, the MHV68 gH protein, the T4 bacteriophage fibritin trimerization domain (foldon), and a 6x His-tag. The gB-mRNA vector encoded a modified MHV68 gB protein, in which the furin cleavage site of the wildtype gB protein was substituted with a flexible (Gly_4_Ser_1_)_3_ linker, flanked by the IL-10 signal peptide and a 6x His-tag. To verify these two vectors, HEK293T cells were transfected with in vitro transcribed mRNA and then analyzed for expression of the target proteins. For the gHgL-mRNA-transfected samples, a ~130 KDa band was detected in the culture medium samples by anti-His-tag antibodies (Figure 1b). Similarly, positive signal was also detected at ~130 KDa for the culture supernatant of the gB-mRNA-transfected samples (Figure 1b). It is noted that the signal intensity of the gHgL-mRNA-transfected samples was much stronger than that of the gB-mRNA-transfected ones (Figure 1b), indicating higher levels of protein expression and secretion for the gHgL-mRNA construct. The molecular mass of the detected positive bands was much higher than the predicted molecular weight (~100 KDa) for both gHgL and gB proteins, suggesting possible glycosylation of the secreted target proteins. To verify this, we performed a protein deglycosylation assay. The supernatant of the mRNA-transfected cell cultures was treated with the endoglycosidases PNGase F or Endo H, and then subjected to Western blotting. The results showed that PNGase F but not Endo H treatment resulted in an obvious decrease in the molecular mass of the positive protein bands (Figure 1c,d), indicating that the secreted gHgL and gB proteins are indeed glycosylated. Collectively, the above data verify the target protein expression of the mRNA vaccine constructs.

### 3.2. The MHV68 gHgL-mRNA Vaccine, but Not the gB-mRNA Vaccine, Induces Neutralizing Antibodies in Mice

**Figure 2:**
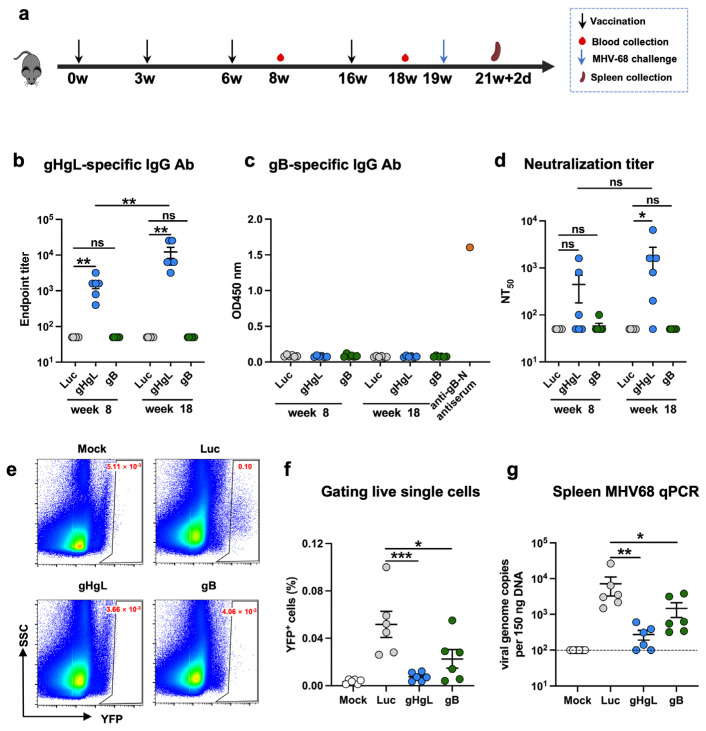
Antibody response and protective efficacy of individual mRNA vaccines in mice. (**a**) Immunization schedule. Groups of C57BL/6 mice were injected i.m. with 10 μg of gHgL mRNA (*n* = 6), gB mRNA (*n* = 6), and Luc-mRNA (Ctrl; *n* = 6) vaccines at weeks 0, 3, 6, and 16. Serum samples were collected from individual mice at weeks 8 and 18. Three weeks after the last immunization, the mice were challenged with 1 × 10^5^ PFU of MHV68-H2bYFP. Spleens were collected on day 16 post-infection. (**b**) Antibody titers to gHgL in immune sera at weeks 8 and 18 determined by ELISA. Binding titer below 1:100 (the lowest serum dilution) was assigned a value of 1:50 for statistical analysis. (**c**) gB-specific antibody response. Immune sera collected at weeks 8 and 18 were analyzed for gB-specific antibody by ELISA with *E. coli*-expressed gB-N protein as the coating antigen. Data shown are OD450 values for individual serum samples. Sera from mice immunized with *E. coli*-expressed gB-N protein served as the positive control in the assays. All serum samples were diluted 1:100 and assayed. (**d**) Neutralizing titers of the week-8 and week-18 antisera samples from each group against MHV68-H2bYFP. Serum samples that exhibited less than 50% neutralization at the lowest serum dilution (1:100) were assigned a NT_50_ value of 50 for statistical analysis. Each symbol represents one mouse. (**e**) Mice were inoculated intranasally with 1 × 10^5^ PFU of MHV68-H2bYFP. Splenocytes were isolated on day 16 post-infection; the flow plot represents the strategy gating YFP^+^ MHV68-infected cells. (**f**) Frequency of YFP^+^ cells on day 16 post-infection determined by flow cytometry. (**g**) DNA was extracted from splenocytes. Viral DNA copy numbers were evaluated via qPCR. Each symbol represents an individual mouse. Dotted line indicates detection limit. For panels (**b**–**d**), data are presented as mean ± SEM. *p*-values were analyzed with two-tailed Mann–Whitney U Test. For panels (**f**,**g**), data are presented as mean ± SEM. *p*-values were analyzed with one-way ANOVA (**f**) and one-tailed Mann–Whitney U Test (**g**), which indicated the following: ns, not significant; * *p* < 0.05; ** *p* < 0.01; and *** *p* < 0.001.

We then assessed the immunogenicity of the MHV68 mRNA vaccine candidates in mice. Prior to immunization, in vitro transcribed antigen-expressing mRNAs were formulated with lipid nanoparticles (LNPs) according to a protocol established previously in our group [50], yielding the MHV68 mRNA vaccine stocks. A firefly luciferase (Luc) reporter-encoding mRNA-LNP formulation was prepared in the same way. Two groups (6 animals/group) of female C57BL/6J mice were immunized intramuscularly (i.m.) with the gHgL-mRNA and gB-mRNA vaccines, respectively, at weeks 0, 3, 6, and 16 (Figure 2a). Another group of mice was injected with the Luc-mRNA/LNP formulation, serving as the control in the experiment. Individual mouse serum samples were collected at weeks 8 and 18 for antibody analysis (Figure 2a). As shown in Figure 2b, all the mice in the gHgL-mRNA group developed gHgL-specific serum antibodies with geometric mean titers (GMT) of 1500 and 12,000 at weeks 8 and 18, respectively. In contrast, none of the serum samples from the control (Luc-mRNA) or gB-mRNA groups exhibited binding activity to the recombinant gHgL protein, even at the lowest serum dilution tested (1:100). We also tested the antisera for the presence of gB-specific antibodies by ELISA with *E. coli*-expressed recombinant gB-N protein (N-terminal half of gB, residues H25 to T430) as the capture antigen. It was found that none of the antisera from the gB-mRNA-, gHgL-mRNA-, or Luc-mRNA-immunized mice showed detectable gB-binding activity, whereas the sera from the mice immunized with *E. coli*-expressed recombinant gB-N protein showed strong reactivity to the capture antigen (Figure 2c). These results indicate that the gB-mRNA vaccine failed to elicit a gB-specific antibody response. Together, the above data demonstrate that only the gHgL-mRNA vaccine elicited MHV68 antigen-specific antibodies in mice.

Next, we tested whether the antisera could neutralize MHV68 infection in vitro. The neutralization assay is based on infection of 3T12 cells with a recombinant YFP-expressing MHV68 virus, MHV68-H2bYFP [45,51]. The results obtained from neutralization assays showed that 50% of the mice in the gHgL-mRNA group developed neutralizing antibodies at week 8, and 83% (5/6) were sero-converted at week 18, with neutralizing titers up to 6400; in contrast, for the gB-mRNA group, only one antiserum sample collected at week 8 showed marginal neutralizing activity (Figure 2d and Supplementary Figure S1). As anticipated, all the antisera in the Luc-mRNA control group failed to exhibit any neutralization even at the lowest dilution tested (1:100) and were therefore assigned a 50% neutralization titer (NT50) of 50 for the geometric mean titer (GMT) computation. For the gHgL-mRNA group, the calculated neutralizing GMTs were 441.7 at week 8 and 1775 at week 18 (Figure 2d).

### 3.3. Both gHgL-mRNA and gB-mRNA Vaccines Are Able to Protect Mice Against MHV68 Infection

We evaluated the protective efficacy of experimental vaccines in a mouse model of MHV68 infection. Three weeks after the last immunization (week 19), mice were intranasally inoculated with MHV68-H2bYFP virus, and 16 days later, mouse spleens were harvested and analyzed for the presence of MHV68-H2bYFP virus (Figure 2a). Flow cytometry analysis revealed that 0.0013% to 0.0051% of the splenocytes from mock-infected mice were positive for YFP, indicating a background level of autofluorescence, whereas the percentages of YFP^+^ cells detected in the Luc-mRNA-immunized, virus-inoculated mice ranged from 0.026% to 0.1%, demonstrating effective latent infection by the MHV68-H2bYFP virus (Figure 2e,f). Notably, the percentage of YFP^+^ splenocytes in the gHgL-mRNA- or gB-mRNA-immunized mice was significantly lower than that of the Luc-mRNA group (Figure 2f), indicating a protective effect of the gHgL-mRNA and gB-mRNA vaccines. To more accurately quantify the virus load, we next performed qPCR analysis to determine the viral genome copy number in individual mouse spleens. As shown in Figure 2g, the mice in the gHgL-mRNA group or in the gB-mRNA group had lower viral genome copy numbers in the spleens compared to those in the Luc-mRNA group. Notably, the viral RNA genome was below the detection limit in two out of six mice from the gHgL-mRNA group (Figure 2g), indicating a sterile immunity against the viral latent infection in a proportion of the vaccinated mice. Interestingly, although the gB-mRNA vaccine-immunized mice did not develop detectable neutralizing antibodies in our assays (Figure 2d), they were, to some degree, protected from latent viral infection, as judged by the significantly reduced viral loads in the spleens (Figure 2g), suggesting that a mechanism other than neutralizing antibody may be responsible for the gB-mRNA vaccine-induced protection.

### 3.4. Both gHgL-mRNA and gB-mRNA Vaccines Elicit Antigen-Specific T-Cell Responses

**Figure 3:**
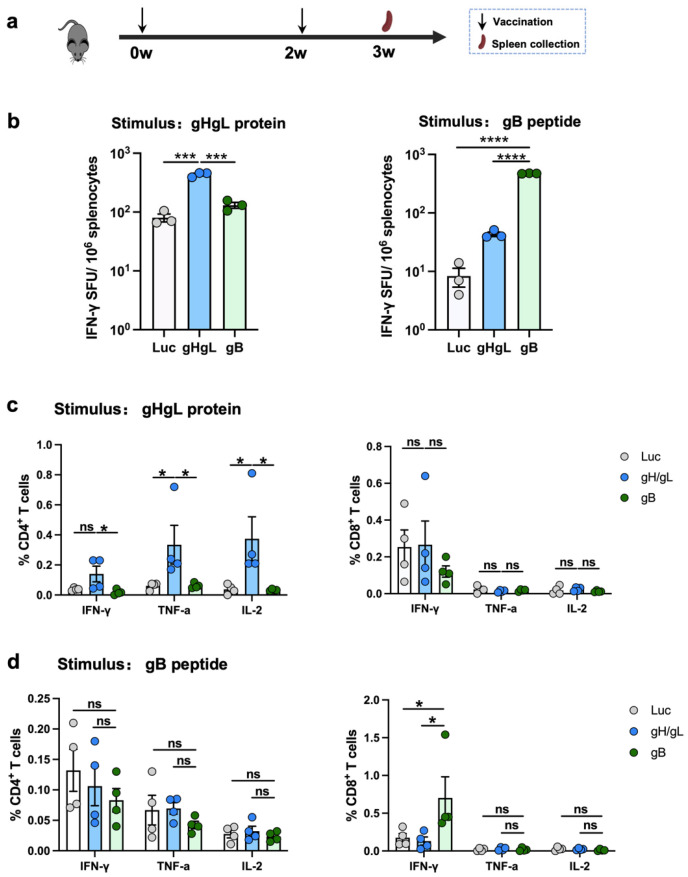
T-cell response elicited by individual mRNA vaccines. (**a**) Immunization scheme. C57BL/6 mice were immunized (*n* = 4 per vaccine group or control group) at weeks 0 and 2 with 10 μg of gHgL mRNA, gB mRNA, gHgL mRNA, and Luc-mRNA (Ctrl) vaccines, and T-cell responses were analyzed at week 3. (**b**) The splenocytes isolated from control or vaccinated individual mice were stimulated with gHgL protein or gB peptide overnight, and gHgL-activated and gB-activated INF-γ^+^ responses were determined by ELISpot assay. The spleen cells from four mice are pooled together. Each symbol represents a repetition. (**c**,**d**) Flow cytometry analysis. Splenocytes were stimulated with (**c**) gHgL protein or (**d**) gB peptide overnight, and the expression of the intracellular cytokines IFN-γ^+^, IL-2^+^, and TNF-α^+^ in CD4^+^ or CD8^+^ T cells was analyzed using flow cytometry. Each symbol represents an individual mouse. For panel (**b**), data are presented as mean ± SEM. *p*-values were analyzed with two-tailed *t*-test. For panels (**c**,**d**), data are presented as mean ± SEM. *p*-values were analyzed with two-tailed Mann–Whitney U Test, which indicated as the following: ns, not significant; * *p* < 0.05; *** *p* < 0.001; and **** *p* < 0.0001.

To assess the cellular immune responses elicited by the experimental vaccines, mice were immunized with two doses of the individual mRNA vaccines. One week after the final immunization, splenocytes were harvested and analyzed using ELISpot and flow cytometry (Figure 3a). In the ELISpot assays, upon stimulation with the gHgL recombinant protein, a significantly higher frequency of IFN-γ-secreting splenocytes was observed in the gHgL-mRNA group relative to the gB-mRNA and Luc-mRNA groups (Figure 3b and Supplementary Figure S2). Similarly, stimulation with a gB-derived peptide (KNYIFEEKL) [52] induced a significantly higher number of interferon (IFN)-γ-secreting splenocytes in the gB-mRNA group compared to the gHgL-mRNA or control (Luc-mRNA) groups (Figure 3b and Supplementary Figure S2).

Subsequently, flow cytometry was performed to further characterize the T-cell responses induced by the vaccines. In the gHgL-mRNA group, a significantly higher proportion of CD4^+^ T cells expressed TNF-α and IL-2 following stimulation with the gHgL recombinant protein (Figure 3c and Supplementary Figure S3). In contrast, upon stimulation with the gB-derived peptide, a significantly higher frequency of IFN-γ^+^ CD8^+^ T cells, but not other T-cell subsets, was detected in the gB-mRNA-immunized mice compared to the gHgL-mRNA and Luc-mRNA groups (Figure 3d and Supplementary Figure S3).

Collectively, these data demonstrate that both the gHgL-mRNA and gB-mRNA vaccines are capable of eliciting MHV68 antigen-specific, functional T-cell responses in mice.

### 3.5. A Bivalent Vaccine Comprising gHgL-mRNA and gB-mRNA Is More Effective than Single mRNA Vaccines

**Figure 4:**
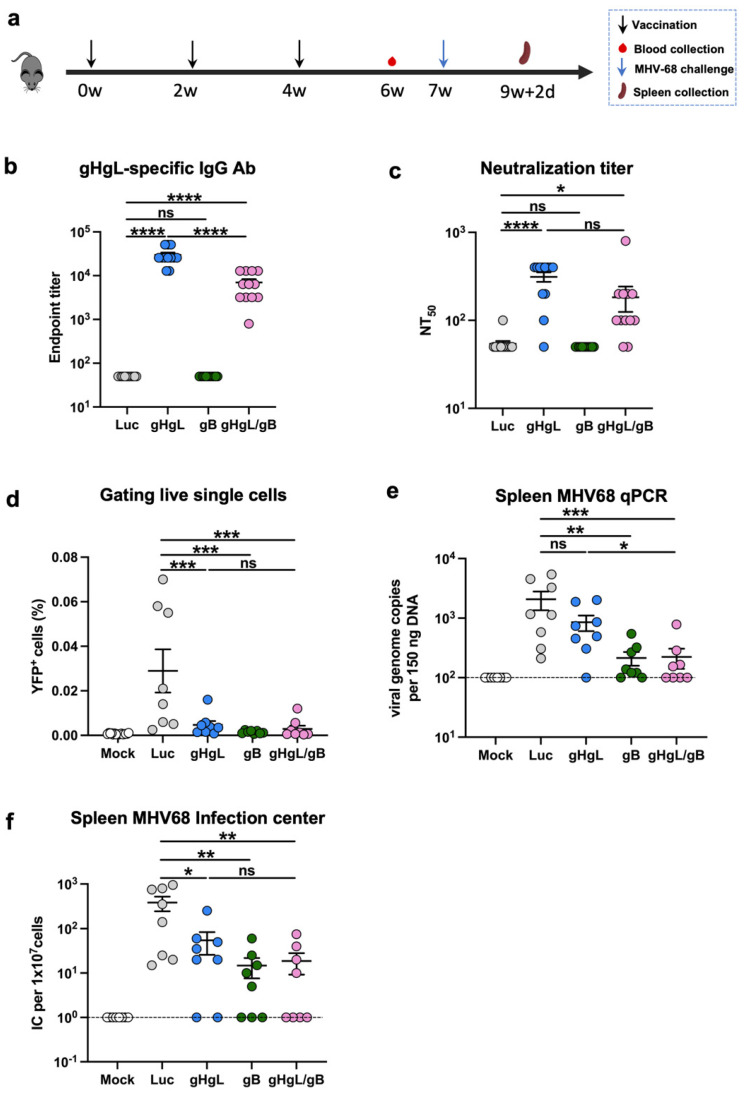
Immunogenicity and protective efficacy of the bivalent mRNA vaccine. (**a**) Immunization schedule. Groups of C57BL/6 mice were injected i.m. with 10 μg of gHgL mRNA (*n* = 12), gB mRNA (*n* = 12), gHgL mRNA and gB mRNA (*n* = 12, 10 μg and 10 μg), or Luc-mRNA (Ctrl; *n* = 12) vaccines at weeks 0, 2, and 4. Serum samples were collected from individual mice at week 6. Three weeks after the third immunization, eight mice per group were subjected to viral challenge. Spleens were collected on day 16 post-infection. (**b**) Antibody titers to gHgL in week-6 immune sera determined by ELISA. Binding titer below 1:100 (the lowest serum dilution) was assigned a value of 50 for statistical analysis. *p*-values were analyzed with two-tailed Mann–Whitney U Test. (**c**) Neutralizing titers of the week-6 antiserum samples from each group against MHV68-H2bYFP. Serum samples that exhibited less than 50% neutralization at the lowest serum dilution (1:100) were assigned a NT_50_ value of 50 for statistical analysis. *p*-values were analyzed with two-tailed *t*-test. (**d**) Frequency of YFP^+^ cells on day 16 post-infection determined by flow cytometry. Each symbol represents an individual mouse. *p*-values were analyzed with one-way ANOVA. Latent infection in the spleens at 16 d post-infection was evaluated by qPCR analysis of viral DNA copy numbers (**e**) and infectious center assay (**f**). Dotted line indicates detection limit. For panels (**b**–**f**), data are presented as mean ± SEM. For panels (**e**,**f**), *p*-values were analyzed with one-tailed Mann–Whitney U Test, which indicated the following: ns, not significant; * *p* < 0.05; ** *p* < 0.01; *** *p* < 0.001; and **** *p* < 0.0001.

Given that individual gHgL-mRNA and gB-mRNA vaccines were somewhat protective in vivo, we next examined whether combining these two vaccines could confer better anti-MHV68 immunity. We formulated a combination vaccine (gHgL/gB-mRNA) consisting of gHgL-mRNA and gB-mRNA in a 1:1 ratio and subsequently compared its efficacy with that of individual mRNA vaccines in a mouse immunization/challenge study (Figure 4a). Groups of C57BL/6J mice were immunized with the gHgL/gB-mRNA combination vaccine (containing 10 μg gHgL-mRNA and 10 μg gB-mRNA), the gHgL-mRNA (10 μg), the gB-mRNA (10 μg), or the Luc-mRNA (10 μg) as the control, respectively, at weeks 0, 2, and 4. Sera were collected from each mouse at week 6 for antibody analysis (Figure 4a). Only mice from the gHgL-mRNA or gHgL/gB-mRNA groups developed gHgL-specific binding antibodies (Figure 4b). None of the antisera from the four mouse groups showed gB-specific binding activity (Supplementary Figure S4), in line with the results of aforementioned experiments (Figure 2a). Next, we determined the neutralizing potency of the antisera. The neutralizing antibody titers in the bivalent vaccine (gHgL/gB-mRNA) group were comparable to those in the single gHgL-mRNA group (Figure 4c). Consistently, only a background level of neutralization activity was detected for the gB-mRNA- or Luc-mRNA-immunized mice (Figure 4c).

The mice were all challenged with MHV68-H2bYFP at week 7 and assessed for latent infection in the spleen after 16 days. The numbers of YFP^+^ splenocytes in the three experimental vaccine groups (gHgL-mRNA, gB-mRNA, or gHgL/gB-mRNA) were close to the background (mock-infected mice) level and were significantly lower than those in the control group (Luc-mRNA; Figure 4d). The qPCR analysis revealed a significant decrease in the viral genome copy numbers in a significant proportion of the gHgL-mRNA-, gB-mRNA-, and gHgL/gB-mRNA-immunized mice in comparison to the control group (Figure 4e). Notably, one out of the eight mice (12.5%) in the gHgL-mRNA group and two of the eight mice (25%) in the gB-mRNA group had no detectable viral DNA, whilst four of the eight mice (50%) in the bivalent mRNA vaccine group were free of viral DNA (Figure 4e), suggesting the absence of MHV68 in these mice. We further performed plaque assays to verify the presence/absence of infectious virus in the mouse spleens. All of the samples that were negative for viral DNA did not generate plaque, confirming that they were indeed virus-free (Figure 4e,f). Altogether, we concluded that the gHgL/gB-mRNA combination vaccine is superior to the single mRNA vaccines in terms of inducing sterile immunity to MHV68 latent infection.

## 4. Discussion

The goal of vaccination against gammaherpesviruses is to prevent latent infection. For MHV68, it has been demonstrated that only vaccine candidates based on replication-competent live attenuated virus are capable of inducing sterile immunity against latent infection [32,33,36]. However, this vaccine strategy is not suitable for human gammaherpesviruses because of potential oncogenic risk associated with live virus. In the present study, we use the MHV68 model to demonstrate, for the first time to our knowledge, that it is feasible to prevent natural gammaherpesvirus latent infection by using mRNA vaccines that target key components of the viral entry complex (e.g., gH, gL, and gB), thus opening up a novel avenue for vaccine development for human gammaherpesviruses.

The MHV68 gH protein is an essential viral protein responsible for membrane fusion during virus entry. It binds to the gL protein to form the gH/gL heterodimer, which is the major target for neutralizing antibodies [43]. In this study, we developed and evaluated a gHgL-mRNA vaccine that encodes a fusion protein between gH and gL. The immunization of mice with the gHgL-mRNA resulted in the production of serum neutralizing antibodies with geometric mean NT50s of 441.7 and 1775 after the third and fourth dose, respectively (Figure 2d). Importantly, following intranasal MHV68 challenge, a proportion (33%) of the gHgL-mRNA-immunized mice was protected from latent viral infection (Figure 2f,g). These data suggest a pivotal role of gH/gL-directed neutralizing antibodies in in vivo protection against MHV68. In addition, the gHgL-mRNA vaccine was able to elicit a gHgL-specific CD4^+^ T-cell response in mice that might have partially contributed to the observed in vivo protection against MHV68 latent infection.

The gB protein of MHV68 is part of the virion fusion complex and possesses neutralizing epitopes [44,53]. Interestingly, our present study shows that the gB-mRNA vaccine did not induce gB-specific antibodies when measured by ELISA with *E. coli*-expressed gB-N protein as the coating antigen. It is possible that *E. coli*-expressed gB-N protein, which lacks eukaryotic post-translational modifications such as glycosylation and therefore does not acquire native conformation, might not be recognized by antibodies raised against mRNA vaccine-produced gB protein. Nonetheless, we found that the gB-mRNA-immunized mice produced no detectable neutralizing antibodies, but they were still protected from viral latent infection to some degree. Consistently, a previous study showed that immunization with vaccinia virus expressing MHV68 gB or its N-terminal part (gB-N, residues 1-423) failed to elicit detectable levels of neutralizing antibody, but it could reduce MHV68 lytic replication in the mouse lung at five days post-challenge [54]. Together, these results suggest a neutralizing antibody-independent protective mechanism for gB. Indeed, we observed that a potent gB-specific T-cell response characterized by IFN-γ^+^ CD8^+^ T cells was elicited in the gB-mRNA-immunized mice (Figure 3b), implicating that gB-based vaccines may confer protection through induction of cellular immune response, particularly CD8^+^ T cells. Further experiments, such as in vivo depletion of CD8^+^ and/or CD4^+^ T cells in immunized mice followed by MHV68 virus challenge, are needed to clarify the role of CD8^+^ and CD4^+^ T cells in gB-mRNA-induced protective immunity.

In this study, we formulated a bivalent vaccine consisting of both the gHgL-mRNA and the gB-mRNA. This bivalent vaccine appeared to more effective than the individual gHgL-mRNA or the gB-mRNA vaccines, judging by the proportion of mice that acquired sterile immunity against MHV68 latent infection. The neutralizing antibody titer of the bivalent vaccine group was similar to that of the gHgL-mRNA group, suggesting that the enhancement in protective efficacy observed with the bivalent vaccine may be attributable to its gB component through the induction of functional CD8^+^ T cells. Hence, our findings demonstrate that both neutralizing antibody and T-cell immune responses play crucial roles in protecting against MHV68 infection, and it is advantageous to elicit a broader T-cell response by targeting multiple antigens.

In summary, our work not only develops a bivalent (gHgL/gB) mRNA vaccine for MHV68 that is immunogenic and proven to be effective in the natural host, but also reveals its protective mechanisms. As the sequence and function of gH, gL, and gB are highly conserved between MHV68 and human gammaherpesviruses (in particular, EBV) [55,56], our findings have the potential to facilitate the design and development of safe and efficacious vaccines against human gammaherpesviruses.

## 5. Conclusions

Our work demonstrates that immunization with mRNA platform-based subunit vaccines is indeed capable of preventing MHV68 latent infection, thus validating a safe and efficacious vaccination strategy that may be applicable to human gammaherpesviruses.

## Data Availability

The data that support the findings of this study are available from the corresponding author upon reasonable request.

## Supplementary Materials

The following supporting information can be downloaded at: https://www.mdpi.com/article/10.3390/vaccines13080830/s1, Figure S1: Representative images of the neutralization assays. Week-6 serum samples from mice immunized with gHgL-mRNA, gB-mRNA, or LucmRNA were tested at a 1:100 dilution for neutralization of MHV68-H2bYFP infection in 96-well plates. Fluorescent foci indicate viral infection of cells. Mock-infected cells served as the negative control. The plates were scanned using the Immunospot^®^ S6 Universal M2 Analyzer, and YFP foci were counted; Figure S2: IFN-γ responses of splenocytes to gHgL protein or gB peptide stimulation measured by ELISpot. Mice were immunized with individual mRNA vaccines as indicated in Figure 3. The splenocytes isolated from control or the vaccinated mice were stimulated overnight with the recombinant gHgL protein or the gB peptide, followed by measurement of IFN-γ-secreting cells by ELISpot assay. Figure S3: Gating strategy for intracellular cytokine staining of CD4+ and CD8+ T cells. (a,b) Intracellular cytokine gating examples are shown from spleen cells of a representative T cell cytokine response to gHgL protein (a) or gB peptide (b). Cells were gated on single lymphocytes based on size, followed by exclusion of dead cells and subsequent gating on CD4 or CD8 subsets. Within each subset, cytokine-producing cells were detected based on intracellular staining; Figure S4: gB-specific antibody response and in vivo protection induced by mRNA vaccines. Mice were immunized with individual or bivalent mRNA vaccines and subsequently challenged with MHV68 as indicated in Figure 4. (a) Measurement of gB-specific antibody responses in the vaccinated mice. Immune sera collected at two weeks after the third immunization were analyzed for gB-specific antibody by ELISA with E.coli-expressed gB-N protein as the coating antigen. Data shown are OD450 values for individual serum samples. Sera from mice immunized with E.coli-expressed gB-N protein served as the positive control in the assays. All serum samples were diluted 1:100 and then assayed. (b) Viral loads detected in the spleen of the immunized mice following MHV68 challenge. 16 days post-challenge, splenocytes were harvested and analyzed for viral load by infectious center assay. For each sample, two concentrations of splenocytes (1 × 10^6^/well and 1 × 10^5^/well) were used for incubation with Vero cells. After five days, the wells were imaged, and plaques were counted. Images of two replicate wells for each treatment are shown.
