# Supplementary material for: A Bivalent mRNA Vaccine Efficiently Prevents Gammaherpesvirus Latent Infection
Source: Vaccines (Basel). 2025 Aug 4;13(8):830. doi: 10.3390/vaccines13080830 (PMC12390394; doi:10.3390/vaccines13080830)
Supplement: Supplementary file 1 [file vaccines-13-00830-s001.zip › vaccines-3730483-supplementary.pdf]

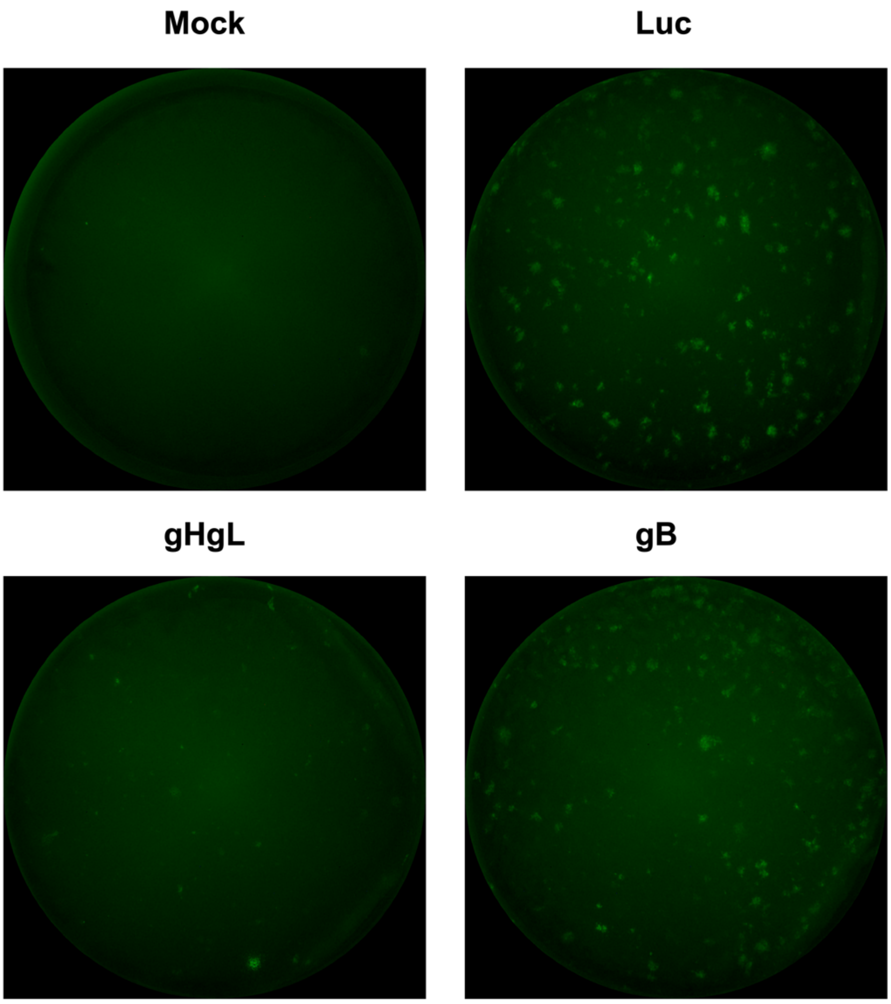

Fig. S1 (relating to Fig. 2). Representative images of the neutralization assays. Week-6 serum samples from mice immunized with gHgL-mRNA, gB-mRNA, or Luc-mRNA were tested at a 1:100 dilution for neutralization of MHV68-H2bYFP infection in 96-well plates. Fluorescent foci indicate viral infection of cells. Mock-infected cells served as the negative control. The plates were scanned using the Immunospot® S6 Universal M2 Analyzer, and YFP foci were counted.

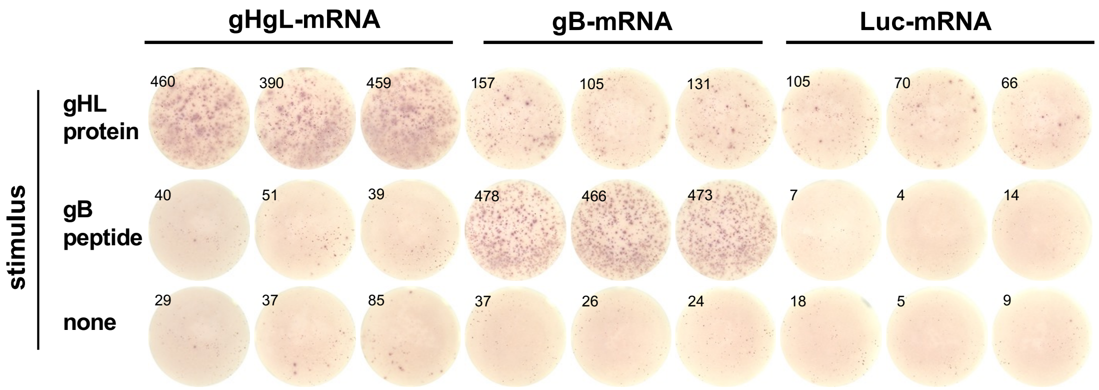

Fig. S2 (relating to Fig.3). IFN-γ responses of splenocytes to gHgL protein or gB peptide stimulation measured by ELISpot. Mice were immunized with individual mRNA vaccines as indicated in **Fig. 3**. The splenocytes isolated from control or the vaccinated mice were stimulated overnight with the recombinant gHgL protein or the gB peptide, followed by measurement of IFN-γ-secreting cells by ELISpot assay.

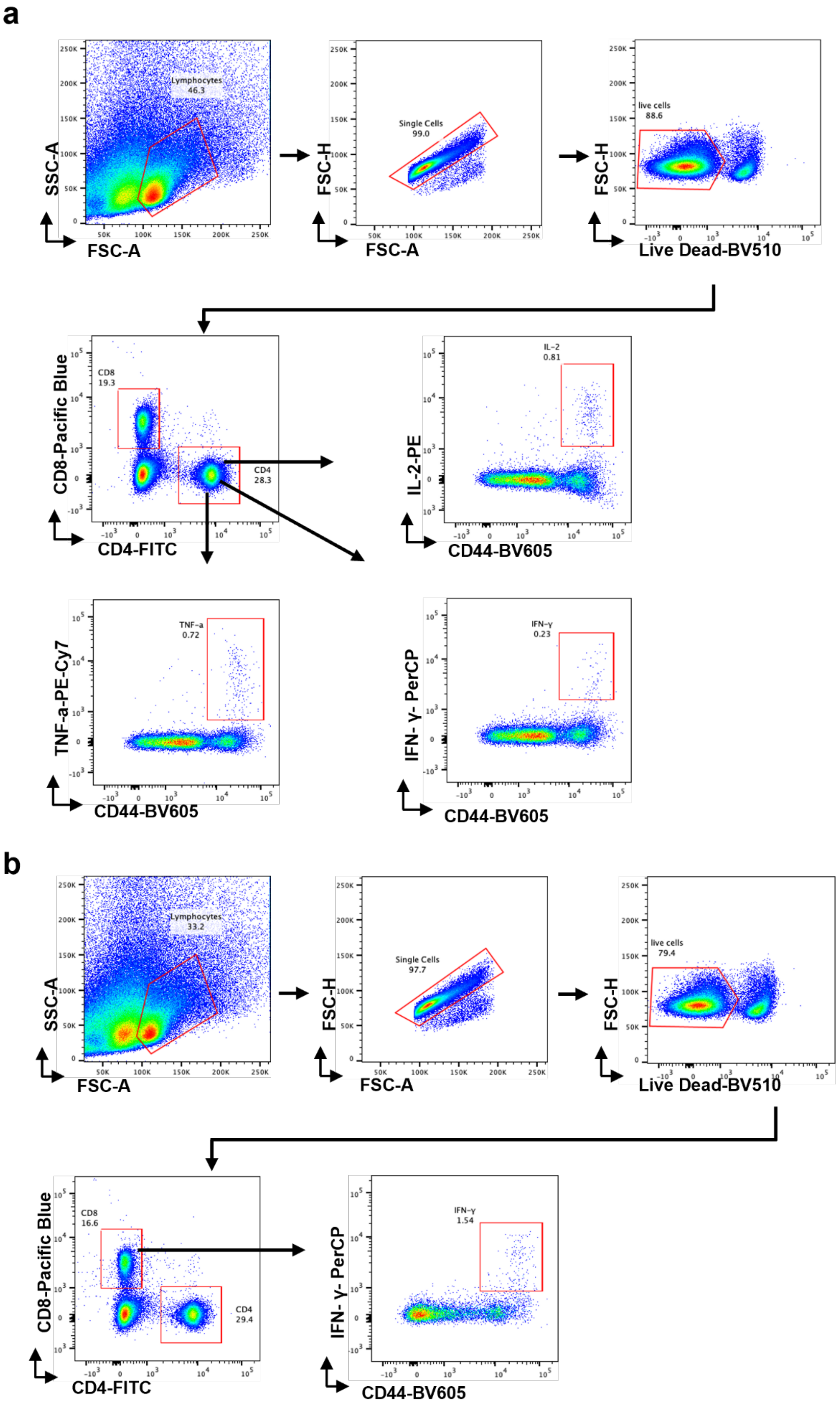

Fig. S3 (relating to Fig. 3). Gating strategy for intracellular cytokine staining of $CD4^{+}$ and $CD8^{+}$ T cells. (a-b) Intracellular cytokine gating examples are shown from

spleen cells of a representative T cell cytokine response to gHgL protein **(a)** or gB peptide **(b)**. Cells were gated on single lymphocytes based on size, followed by exclusion of dead cells and subsequent gating on CD4 or CD8 subsets. Within each subset, cytokine-producing cells were detected based on intracellular staining.

a

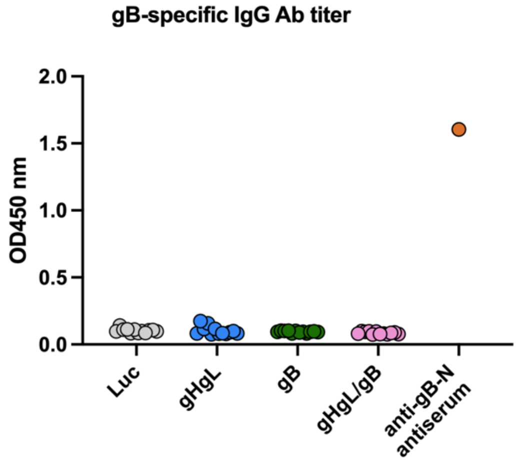

b

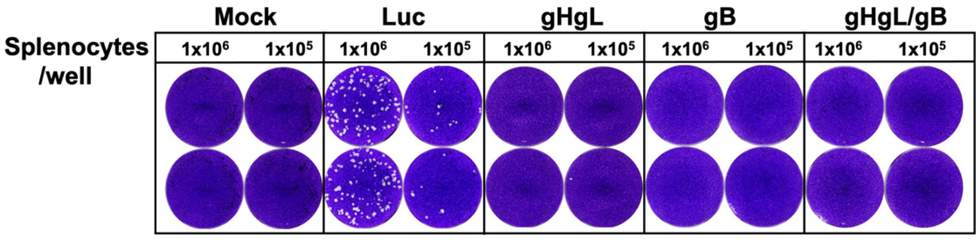

Fig. S4 (relating to Fig. 4). gB-specific antibody response and *in vivo* protection induced by mRNA vaccines. Mice were immunized with individual or bivalent mRNA vaccines and subsequently challenged with MHV68 as indicated in **Fig. 4**. **(a)** Measurement of gB-specific antibody responses in the vaccinated mice. Immune sera collected at two weeks after the third immunization were analyzed for gB-specific antibody by ELISA with *E.coli*-expressed gB-N protein as the coating antigen. Data shown are OD450 values for individual serum samples. Sera from mice immunized with *E.coli*-expressed gB-N protein served as the positive control in the assays. All serum samples were diluted 1:100 and then assayed. **(b)** Viral loads detected in the spleen of the immunized mice following MHV68 challenge. 16 days post-challenge, splenocytes were harvested and analyzed for viral load by infectious center assay. For each sample, two concentrations of splenocytes (1 x 10^6/well and 1 x 10^5/well) were used for incubation with Vero cells. After five days, the wells were imaged, and plaques were counted. Images of two replicate wells for each treatment are shown.
